# Infusing disability equity within rehabilitation education and practice: A qualitative study of lived experiences of ableism, allyship, and healthcare partnership

**DOI:** 10.3389/fresc.2022.947592

**Published:** 2022-08-02

**Authors:** Heather A. Feldner, Heather D. Evans, Katherine Chamblin, Lesley M. Ellis, Mark K. Harniss, Danbi Lee, Joanne Woiak

**Affiliations:** ^1^Department of Rehabilitation Medicine, University of Washington, Seattle, WA, United States; ^2^Disability Studies Program, University of Washington, Seattle, WA, United States; ^3^Center for Research and Education on Accessible Technology and Experiences (CREATE), University of Washington, Seattle, WA, United States; ^4^The Disability and Deaf Cultural Center, University of Washington, Seattle, WA, United States

**Keywords:** disability studies, rehabilitation, equity, ableism, allyship, qualitative inquiry

## Abstract

**Background:**

Addressing issues of diversity, equity, and inclusion (DEI) has become central in implementing inclusive and socially responsible rehabilitation education and clinical practice. Yet, the constructs of disability and d/Deaf identity and culture, as well as ableism and allyship are often overlooked. Or, these concepts are approached using outdated philosophical perspectives that pathologize disability and fail to prioritize the lived experiences, expertise, intersectionality, and self-identified needs of people with disabilities. A Critical Disability Studies (CDS) framework may provide a background for better understanding and responding to these issues through allyship.

**Purpose:**

This study employed a CDS framework to understand the lived experiences of ableism and allyship from faculty, staff, and students on University of Washington (UW) campuses who identify as d/Deaf, disabled/with a disability, or as having a chronic health condition.

**Methods:**

During 2020–2021, we conducted in-depth, semi-structured interviews and focus groups with 22 diverse undergraduate and graduate students, faculty, and staff with disabilities, one third who also identified as people of color. Encounters were audio-recorded, transcribed verbatim, and coded using constant comparison until themes emerged.

**Results:**

Four major themes that emerged from the data are: (1) Ever-present ableism in healthcare, (2) Ableism at the intersections, (3) COVID: Surfacing ableism and expanding access, and (4) Disability allyship and healthcare partnership building. Experiences of ableism and allyship were identified at individual, group/unit, and institutional/systemic levels, though participants reported significantly fewer instances of allyship compared to experiences of ableism. Participants identified intersections between disability and other marginalized identities and juxtaposed the benefits of widespread adoption of many access-increasing practices and technologies due to the COVID-19 pandemic, while also highlighting ways in which the pandemic created new obstacles to inclusion.

**Conclusions:**

This analysis provides insights into ways of implementing inclusive practices in rehabilitation education, practice, and beyond. Rehabilitation students, faculty, and staff may not be aware of how ableism affects their disabled peers or underpins their professional education. It is important to cultivate opportunities within professional education and clinical training to explicitly address our collective role in creating inclusive and accessible academic and healthcare experiences for our diverse community post COVID-19. Drawing on a CDS framework, the research team devised the mnemonic TRAC, which includes Training, Recognition and Representation, Attendance and Action, and Calling to account as strategic guidelines for operationalizing such opportunities.

## Introduction

Disabled people have long had disparate access to care and community. This has been exacerbated by the COVID-19 pandemic due to isolation, loss of services, and heightened risk of infection for immunocompromised individuals and those who remain at increased risk, even as non-disabled people return to their routines as the virus reaches endemic phases (1–3). Emerging research with disability communities during this period has addressed critical barriers that disabled people continue to face, with momentum building toward a re-envisioned future that includes greater support of employment and remote work for disabled people, improving disability-inclusive public health responses and rehabilitation practices, and shifting society's messaging about health, disability, and intersectionality post-pandemic (4–9). Given this re-envisioning, there is both a unique opportunity and responsibility in the rehabilitation field to engage in translational research that foregrounds the lived experiences of disabled people in all aspects of life during and after the pandemic. In particular, it is essential to critically examine how disability can be recognized and included in current and future diversity, equity, and inclusion (DEI) practices as a central component of implementing inclusive and socially responsible practices in contemporary rehabilitation education and practice.

Disability is a ubiquitous and intersectional human experience, and there is a critical need to include disability in DEI initiatives. Creating and sustaining a culture that embodies DEI within institutions of higher learning and their surrounding communities has become an increasingly urgent priority across the US (1). As a part of this priority, identifying and addressing barriers to equity and justice are essential in more effectively implementing inclusive and responsible social, educational, and administrative practices on college campuses, and in professional programs that train future rehabilitation practitioners (2). Yet, while the National Center for Education Statistics indicates that nearly 20% of undergraduate students and 12% of graduate students report having some type of disability (3), the constructs of disability, d/Deaf or disability identity and culture, and allyship are routinely overlooked in DEI initiatives (4). When disability *is* included, it is often considered from outdated philosophical approaches that pathologize disability, focus primarily on educational accommodations, and fail to prioritize the lived experiences, expertise, and self-identified needs of people with disabilities (5, 6). This can be harmful and unwelcoming when a perspective of “normalizing,” “fixing,” or “overcoming” disability is presented in classroom narratives or materials, or when physical spaces (i.e., exam rooms, classrooms) lack adjustable or modular equipment. Further, there is a lack of acknowledgment or discourse surrounding intersectionality, which underscores the juncture of multiple marginalized identities that lead to experiences of systemic oppression, including how disability may intersect with race, gender, socioeconomic status, sexuality, and citizenship status (4, 7, 8). Many scholars of color have called for greater attention specifically to the intersection of disability and Blackness, both before, and as a result of, the widespread Racial Justice protests of 2020, the Black Lives Matter movement, and disparities made plain by the COVID-19 Pandemic (9–15). Momentum is building to ensure that these critical topics are threaded throughout higher education, and especially within professional rehabilitation education to foster the development of inclusive healthcare practitioners (16).

Many of the problematic views toward disability, as well as the failure to include disability as an integral part of DEI initiatives, have to do with deeply rooted societal ableism. Though nuances exist in defining “ableism” or “disableism,” operationally we refer here to the normalized preference for certain abilities and sustained discrimination against and oppression of people with ways of being, functioning, and in some cases, simply appearing (such as individuals with facial scarring) that are viewed as non-typical by the sociocultural norms of a given society (17–19). Ableism thus encompasses prejudice, stereotypes, and bias against disability at the individual level as well as the institutionalization of systemic advantages and privileges granted to those whose bodies and minds conform to societal expectations, creating what some scholars have described as a “compulsory preference for non-disability” (20–24). Like in efforts to dismantle racism or sexism, social justice advocates have pointed to the need for structural changes at the institutional and societal levels as well as the spread of disability allyship at individual and group levels for meaningful change to occur.

The process of allyship, as a series of actions to recognize and mitigate discriminatory practices, has been touted as a direct means of challenging, or subverting ableism. While terms are still contested within the disability justice space (i.e., ally vs. accomplice), disability scholars have described allyship as the amplification of disabled voices and experiences in all aspects of society, meaningful engagement within disability communities in solidarity, engaging in inclusive practices, rejecting performative allyship, rejecting harmful disability narratives, becoming educated about disability oppression, and leveraging privileged positionalities to call out ableism and injustice and produce actionable change (25–28). Scholars in rehabilitation have contributed as well, describing how rehabilitation professionals can practice allyship with disability communities by playing a supporting role in their clients' disability identity journey, educating themselves about intersectionality, honoring preferred language, and engaging in advocacy outside clinical settings (29–33). Further, this work acknowledges the power differential typically experienced by practitioner and client, and pushes back against assumptions frequently made by practitioners that disability is inherently viewed as a “problem” by their clients, or that their role is one of “helper” or “saver” rather than collaborative partner (29, 33). Disrupting and addressing these assumptions is central to efforts to dismantle ableism, and these strategies must be concretely implemented as a form of allyship in rehabilitation education and practice.

**Figure 1:**
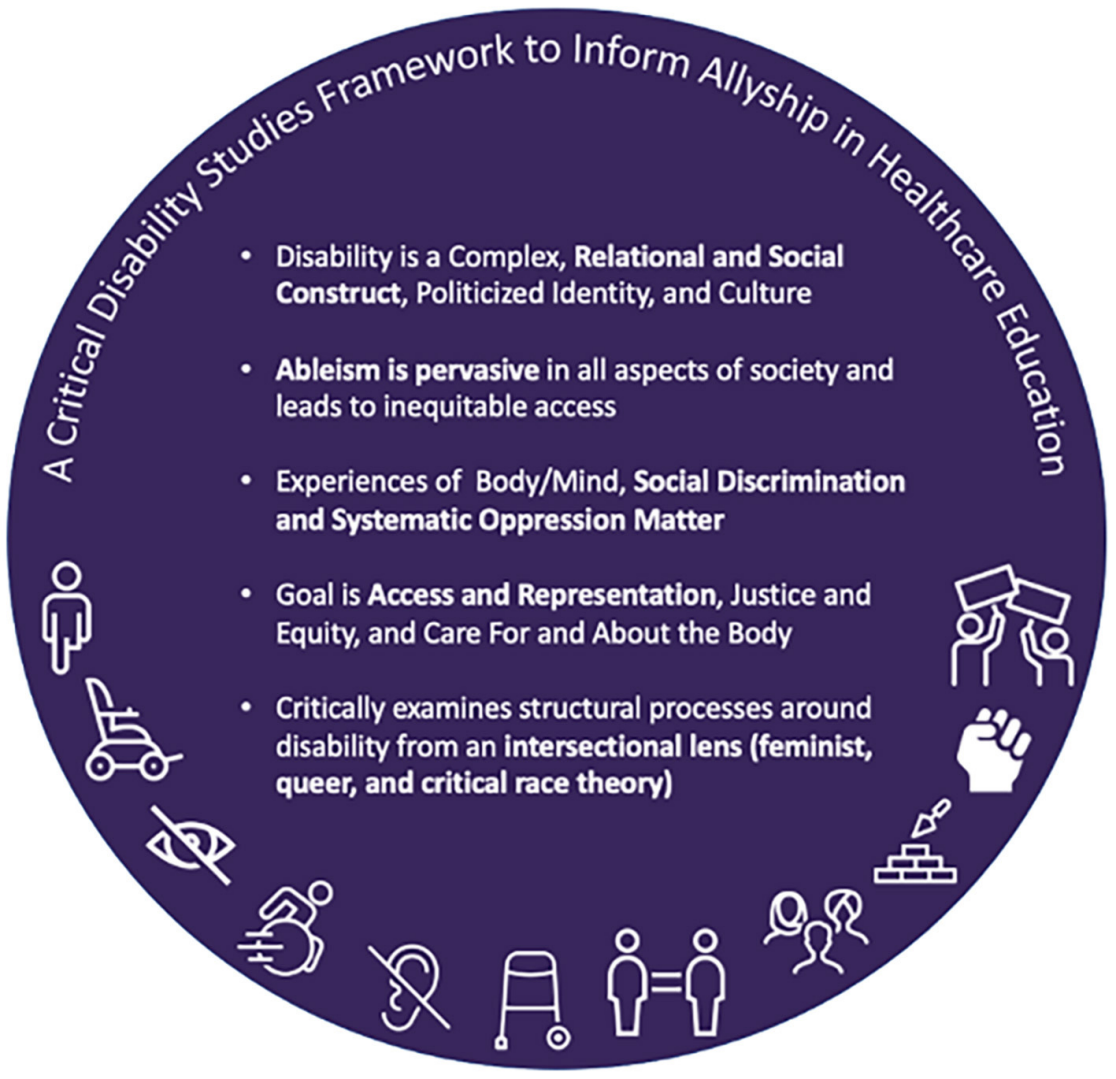
A list of five core tenets comprising a critical disability studies framework to inform allyship in healthcare education.

A Critical Disability Studies (CDS) framework (Figure 1) can provide the background for understanding and responding to these issues, and facilitate more explicit inclusion of disability in DEI initiatives through allyship and activism. CDS foregrounds the lived experiences of people with disabilities, recognizes and interrogates the physical, attitudinal, socio-economic, and institutional discrimination and barriers faced by people with disabilities in our society, and offers a critical lens through which to evaluate the meanings of disability and allyship, as well as the processes and practices within higher education and across campus culture (6, 34–36). A CDS approach recognizes disability as a complex, relational, social and political identity as well as minority culture. It offers shared importance to the experiences of the body/mind and the need for care and attention to the body/mind, to address pain, the need for medication or assistive technology, or other health and wellness necessities. However, CDS also interrogates the social and political environments in which disabled people exist, calling attention to social discrimination and oppressive structures and practices rooted in ableism. CDS recognizes that both these aspects of disability matter, and must be addressed through access, representation, and justice. This approach is especially useful in rehabilitation and healthcare fields, which have been historically focused on restoring the function of an able body. However, aside from a few notable exceptions, among them Gibson's seminal book *Rehabilitation: A Post-critical Approach*, a CDS perspective has been largely absent in rehabilitation to date, especially in the United States (US) (16, 37–39). Further, while disability representation is increasingly espoused is these fields, it remains critically low (40–42).

The purpose of this study was to employ a CDS framework to understand the lived experiences of ableism and allyship among faculty, staff, and students on University of Washington (UW) campuses who identify as d/Deaf, disabled/with a disability, or as having a chronic health condition. These findings provide insights into ways of implementing inclusive practices in rehabilitation education, practice, and beyond.

## Methods

This was a qualitative study conducted from a phenomenological perspective, employed to describe and understand the shared and unique lived experiences (phenomena) of students, staff, and faculty on the UW campuses who self-identify as disabled/with a disability, d/Deaf, or having a chronic health condition. Phenomenology is an analytic perspective focusing on understanding the essence or core set of shared experiences of individuals involved in a particular phenomenon, in contrast to techniques that solicit opinions about issues or analyze actions in experimental or hypothetical situations (43–46).

All research activities involving participants were conducted after receiving approval from the UW Human Subjects Division and all participants provided verbal informed consent for their participation prior to engaging in any research activities.

### Positionality of the researchers

The multidisciplinary research team has both lived and professional expertise with disability and disability-related issues, consisting individuals (5 women and 1 man) who identify as disabled and non-disabled. Professionally, two of the authors are rehabilitation professionals (one occupational therapist and one physical therapist), one is the Director of the UW Deaf and Disability Cultural Center, four are employed within UW's Department of Rehabilitation Medicine, and five are core faculty in the Disability Studies program at UW. These lived and professional experiences led to a diverse and representative research team but also contributed potential biases to the development and interpretation of this study, given the potential nuances of individual disability experiences, language preferences (i.e., person-first vs. identity-first language), and academic background. Care was taken during the development of the research materials to examine and mitigate these potential biases through discussion, debate, and collaborative decision-making, writing and editing among the entire research team. To reflect the preferences of our disabled colleagues and participants, identity-first language is used throughout this manuscript (18, 47, 48).

### Participants

We recruited individuals across UW campuses and units using emails and flyers distributed to known campus groups, units, and student organizations, listservs, and faculty newsletters. We employed snowball sampling combined with purposive sampling to maximize racial diversity among our participants with disabilities, as well as ensure representation from a wide range of roles (i.e., student, faculty, and staff) (49). To do this, the research team intentionally posted recruitment flyers to student organizations serving minority students, including students of color. Inclusion criteria for the study were: (1) age 18 or over; (2) self-identify as disabled/with a disability, d/Deaf, or having a chronic health condition; (3) at least a part-time position in a staff, faculty, undergraduate/graduate student role, or recent UW alumni; and (4) be able to proficiently participate in an interview or focus group using English (verbal or *via* communication device), or American Sign Language (ASL). Potential participants self-selected to contact the research team and all underwent screening to ensure inclusion criteria were met, including providing informed consent. Participants were reminded throughout the process that the discussion guide did not ask for details regarding specific impairment and that any such information could be voluntarily shared but was not required. To protect privacy and confidentiality related to disability disclosure, participants were able to self-select whether they preferred to attend an individual interview or focus group. All participant names have been replaced with pseudonyms.

### Procedures

#### Data collection

This study was completed during the COVID-19 pandemic. From 2020 to 2021, we conducted virtual semi-structured interviews and focus groups, each lasting between 1 and 2 h, with cohorts of graduate and undergraduate students, staff, and faculty across two UW campuses. An interview and focus group guide were created by the research team and vetted among members of the disability community not affiliated with the study to target three main topic areas, including Experiences of Ableism, Experiences of Allyship, and Healthcare Experiences. Due to the complexity of individual conceptualizations of disability, ableism, and allyship, working definitions of these terms as defined by individuals in disability communities were included in the guide. The full interview or focus group guide was shared in advance of the interviews as an access measure, and is included in Appendix A in Supplementary material. All interviews and focus groups took place virtually with two facilitators from the research team, and between one and eight participants. Because both the research team and participant pool are part of the same university, facilitators were assigned to avoid colleagues interviewing colleagues and instructors interviewing current students. A script was used to ensure consistency in question introduction and delivery among the research team, and at least one facilitator kept handwritten notes from each session as an audit trail (43). Accommodations such as ASL interpreters and captioning were provided based on participant needs. Each virtual session was audio-recorded.

As discussed in the positionality statement above, the research team represented an interdisciplinary set of scholars including those identifying as disabled as well as non-disabled with training in the humanities, social sciences, and medical fields. In order to concretize our focal constructs and provide a shared starting point for discussion with participants, the collaborative team co-constructed a comprehensive discussion sheet of the key concepts of the study. Given the expansive range of meanings currently assigned to terms such as “disability” or “allyship,” the study team shared with participants, prior to meeting with them, the discussion sheet of the way in which this project approached these terms. Facilitators shared their individual positionalities and reviewed the discussion sheet at the start of each interview or focus group. The text shared with participants explaining the research team's use of disability, ableism, allyship as constructs is provided in full below.

**Disability** is understood to arise from the interaction between a person's health condition or impairment and the multitude of influencing factors in their environment. Many people also refer to themselves as a person with a disability, being disabled, having impairments or health conditions, or by a specific medical diagnosis or cultural group such as autistic, blind, D/deaf, linguistic minority. It is important to recognize that many people believe that disability is NOT a trait or attribute of the individual; rather, disability is experienced as a result of levels of structural oppression based on ideologies that denigrate “disability.” Many disabled people attribute external prejudice and denigration in social and cultural spheres as one of the reasons they experience “disability.” They challenge the idea of “typical functioning” of both the body and mind as a measure of ability or inability. The failure of society to recognize differences in functioning of the body/mind as both natural and neutral is the root cause of “disability.” Disability intersects and is connected to all forms of structural oppression such as racism and sexism, especially those rooted in Western ideologies of white patriarchal supremacy and colonial histories of domination. Disabled people experience disability in different ways depending on other aspects of their identity and lived experience. It is acknowledged that the study of disability, and historically disability research, is also rooted in these structures of power.

**Ableism** is a set of assumptions and practices promoting the differential or unequal treatment of people because of actual or presumed non-typical functioning (i.e., disability). Ableism takes many forms, including non-disabled people controlling disabled people's narratives, judgements on the reality and quality of disabled people's lives, and assumptions that disability is static or unchanging. It is important to note that Ableism is NOT just directed at bodies/minds with physical impairments/and “apparent” differences. People with non-apparent impairments navigate ableism in both similar and different ways. Through this research project, we are hoping to explore the many ways ableism affects individuals with chronic illness, psychiatric impairments (mental illness), learning impairments, physical impairments, and internalized ableism.

**Allyship** is a process rather than a singular concept; allyship consists of a series of actions, and approaches that attempt to recognize, mitigate and challenge structural forms of oppression toward specific communities within both interpersonal interactions and in systemic changes to existing power inequalities. Practicing allyship with and for disabled people/PWD is continually checking assumptions about what informs your views about disability and ability. Some of the ways allyship toward the disability community is practiced is by:

- Listening to disabled people's stories
- Educating yourself about disability and current issues that impact their communities
- Checking assumptions about who is non-disabled/disabled
- Challenging cultural narratives that reinforce ideals of normality
- Identifying and challenging ableist terminology.

#### Data analysis

**Figure 2:**
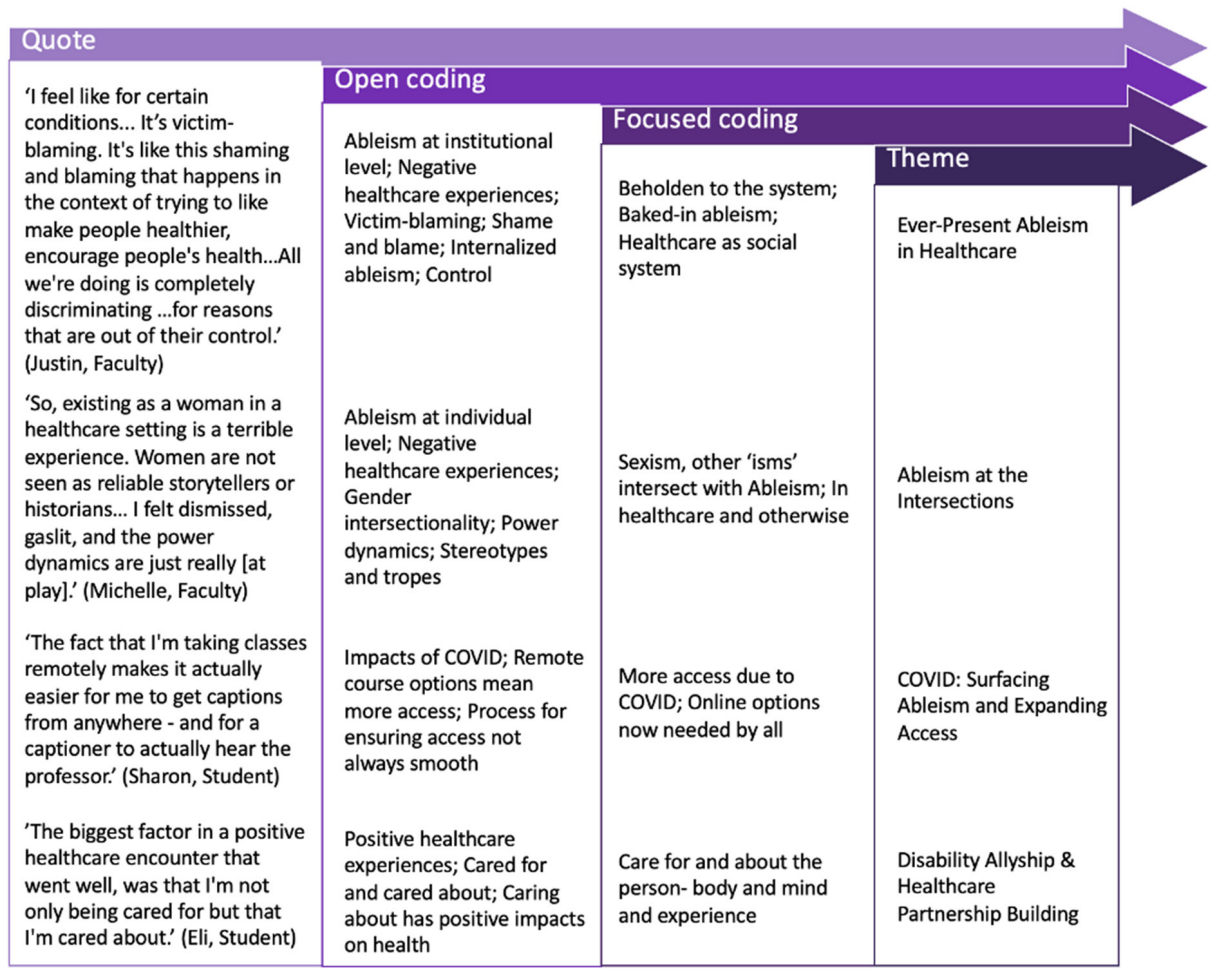
Diagram of three-step coding process showing how individual participant quotes were categorized through open coding, then focused coding, and finally aggregated into four dominant themes. Four example quotes are provided listing each quote's open coded category, focused coded category, and respective theme.

All audio recordings were transcribed verbatim. Transcripts were analyzed using DeDoose Qualitative Analysis software (Hermosa Beach, CA) until data saturation was reached and outliers were identified. All members of the research team engaged in independent coding, with two authors independently reviewing and discussing all coded transcripts. A constant comparison coding procedure was used to first create open codes, followed by focused codes (43). Focused codes were then grouped into themes and discussed amongst the analysis team until consensus was reached. Figure 2 depicts the coding process created as part of our audit trail.

A summary of the themes was shared with participants for review to ensure accuracy and to avoid misinterpretation of the data. Participants also were provided an invitation to engage in member checking, by reviewing their own de-identified session transcript, if desired, and were invited to participate in subsequent research activities, such as the creation of curricular modules for use in allyship training based on the aggregate results from this qualitative study.

## Results

Twenty-two disabled participants (4 men, 18 women, 0 others) across two of the three UW campuses took part in the study, over one-third of whom identified as individuals of color (*n* = 7). Ten were undergraduate students, seven were graduate students, two were staff, and three were faculty. Four participants across roles were engaged in healthcare education, service delivery, or administration. No refusals or withdrawals from the study occurred.

Major themes that emerged from the data included (1) Ever-present ableism in healthcare, (2) Ableism at the intersections, (3) COVID: Surfacing ableism and expanding access, and (4) Disability allyship and healthcare partnership building. These themes encompass participants' experiences in healthcare, which included instances of both allyship and ableism, as well as specific solution building for greater equity in healthcare. Participants juxtaposed the benefits of widespread adoption of many access-increasing practices and technologies due to the COVID-19 pandemic, while questioning whether expanded access will remain when pandemic concerns wane.

Though clear instances of partnership building were identified, participants had numerous negative encounters in healthcare contexts, ranging from communication concerns, inaccessible clinic or exam space, challenges with transportation or other accommodation, and ablest microaggressions. In the following section, we explore each of these themes in more depth.

### Ever-present ableism in healthcare: “They think of you as a train wreck”

Disabled faculty, staff, and students reported experiences of ableism and allyship at individual, group/unit, and institutional/systemic levels, though participants reported significantly fewer instances of allyship compared to experiences of ableism. When describing negative healthcare experiences, numerous participants reported having their symptoms minimized, dismissed, or directly disbelieved.

Notably, respondents discussed ways in which some medical training techniques intended to sensitize healthcare providers to experiences of disability or chronic illness actually led doctors to minimize the lived experiences of their patients. Participants described how providers cited or shared short-term “simulation” experiences in well-meaning but misguided and ableist attempts to empathize with their lived experiences. For example, one participant with Type 1 Diabetes noted:

*I had an Endocrinologist, who chose to be a Diabetes doctor, make a comment to me that she wore an insulin pump for one day, so she knows exactly what it's like to wear one and manage Diabetes… I guess in a way [it felt like she was] almost dismissing the challenges that I go through every day that are hard. That was pretty bad. (Justin, Faculty)*

Another participant described attempts to proactively seek services and being dismissed:

*Another [negative encounter] that comes to mind, is actually the first primary care provider that I went to, about getting diagnosed with Generalized Anxiety. So, I went to another doctor and I came in and I'm like, “I'm having panic attacks.” I'm like, “I can't be in a room full of people anymore. I am having a really hard time being in crowded rooms and being in classes. It's really hard for me.” He was like, “Oh, that's kind of normal.” He seriously said something like, “It's normal for when you get older, you're just more aware of these things. You're okay. Just try to breathe through it. You're fine.” Things like that. I had to basically be like, “Can you refer me? Can you just get me to a psychologist?” (Amanda, Graduate Student)*

Amanda continued her reflection on the incident, directly linking physicians' quick dismissal of particularly mental health symptoms to not just the perpetuation of ableism, but to internalized ableism experienced by disabled people themselves. She continued:

*I know enough about this, that I'm like, “All I need is for you to write a referral, so that I can get out of here.” Like luckily, I know that. But people who don't know that, are probably just like, “Oh yeah, I'm fine. That's fine. You're right.” So, I think yeah, thinking back on that experience, I'm like, “Whoa, that was not okay and that was definitely a really negative experience for me”, as like the first time me confiding in a healthcare professional and them dismissing it. That was not a good experience and I think that that promoted internalized ableism, for sure. (Amanda, Graduate Student)*

Many participants discussed having their earned-through-lived-experience expertise on their condition and their bodies dismissed or challenged outright. In some cases, this resulted in receiving less than optimal treatment and led numerous participants to cycle through providers in search of adequate care. One participant provided an example of having to argue with a physician on a progressive treatment for her condition:

*So, he said, like, ‘Oh no no no, that [treatment] is not an option', but I know that it is an option, I'm in, like, support groups for my disease, and I know that people have done [this treatment] in the past. And so, if I had been more poorly educated then that would have been the end of it, you know, and I would have just gone on, because of that person's biases… And so that left like a really bad taste in my mouth and I switched providers. (Delilah, Staff)*

Notably, a number of participants who are medical professionals themselves described how ableism in healthcare manifests as “intolerance” for disabled people, whether this was related to perceptions of deficit or paternalistic decision-making and communication. One participant noted:

*One thing I found most shocking, ever since I got interested in medicine and started shadowing and volunteering, and then as a med student, I just have been unbelievably* ***shocked by how intolerant of chronic illness and disabilities most physicians really are****. It's really kind of shocking that they… it's sort of like they don't have… the patience or the understanding and that I just do not get. I've tried to figure that one out and it's almost like, I don't know, like it's somehow, you're defective… I don't know why they would react that way because you would think it would be they're the people that would be embracing you and the most understanding…* ***Some doctors just hate complex patients*** ***and you know they think of you as a train wreck****, right? And so, they just don't want to deal with it, they don't, a lot of them don't know enough about these things because, like my stuff is, can be so rare that they don't really know what they're doing but they sort of have that MD attitude thing and then they just decide, so they just totally discount what I say and that's when I really end up in trouble, you know, where I'm desperately sick and can get hospitalized. (Jeanette, Staff)*

Another stated:

*Sometimes it's an academic medical setting where they are trying to teach, and so they're talking more to the resident than they are to me and so I have some patience for that, having been a trainee. But I'm paying to be here and the many, many times I've been told... I mean [I was] flat out, at 25, told, “You're inexplicably disabled and we don't know why but you're never going to work again. And, “Bye”. No, I won't see you again, right. (Michelle, Faculty)*

Another medical professional described the “shaming and blaming” that frequently occurs in the context of health promotion for disabled people, stating:

*I feel like for certain conditions... it's that kind of victimizing, victim blaming. It's like this shaming and blaming that happens in the context of trying to like make people healthier, encourage people's health. And you're like, “This is not productive at all”. All we're doing is completely discriminating and throwing these groups of people under the bus for reasons that are out of their control. (Justin, Faculty)*

Participants who use assisted or alternative forms of communication consistently described having not only their embodied expertise dismissed, but their voices ignored altogether. A young trainee in human services succinctly described the paternalistic practices embedded in health care, stating:

*They don't think people with disabilities can make [the] right decision, so they don't ask me. They ask caregivers instead. There's this bias - that people with disabilities are babies. (Sung-Ho, Graduate Student)*

### Ableism at the intersections

While most participants reported experiencing discrimination and microaggression based on their disability, for those with intersecting marginalized identities, these experiences were exacerbated by sexism, racism, non-citizenship status, and/or fatphobia (weight shaming). Here, intersections are defined as Crenshaw first described: a means of understanding how race, class, gender, ability, and other characteristics overlap and intersect to influence an individual's lived experiences (7).

Several participants identifying as women reflected on how their gender identity influenced their experience of disability in healthcare encounters. One participant explained:

*So, existing as a woman in a healthcare setting is a terrible experience. Women are not seen as reliable storytellers or historians and so, in 90% of my healthcare experiences, I felt dismissed, gaslit, and the power dynamics are just really [at play]. (Michelle, Faculty)*

One participant, also discussing the difficulty of untangling the impact of gender vs. disability bias in her healthcare education experiences, explained that she has become so used to sexist microaggressions that she is slow to recognize—and to call out—ableist microaggressions:

*I think the whole idea of gender being a big part of it too. For me, my anxiety really impacts me in just daily life and being in crowds and things like that, is really hard for me and so I think a lot of times, professors just kind of see that as like, ‘Well she's a woman, so she's emotional. Well, she's a woman, so she's this, that, the other.' So, I think I had definitely internalized that. And I think that that's a lot of what my reaction to the situation in the [lab course] was… Like, my [male student] friend…[was] just blatantly disregarding my disability. … But I think that that was ableism and I really did not realize it, because I was like, ‘Well, I'm so used to people just having sexist microaggressions toward me', that that's just what it felt like and I was like, ‘Okay, whatever'. (Amanda, Graduate Student)*

Amanda's comment demonstrates how multiple forms of oppression shape her relationships with both instructors and fellow students in medical school. For example, she has become so accustomed to people attributing her discomfort in social situations to the gendered stereotype that women are “so emotional” or having male colleagues interrupting or speaking over her, that it has made it challenging for her to get her accommodation needs taken seriously by professors or to assert, even with peers, her need for space to process information and engage at her own pace.

Participants describing themselves as biracial, Indigenous and/or people of color shared negative experiences in both educational and medical contexts that they perceived to be based on disability intersected with another marginalized identity. Numerous participants reported interactions they felt reflected a combination of disability stigma and racial bias, or prejudices against individuals with low socioeconomic or immigrant status. A medical student juxtaposed her healthcare interactions with American doctors vs. those in other countries:

*Again, I think in the US my healthcare interactions were very much colored by color. I overwhelmingly feel blame, I feel shame, I feel between the lines of what providers say. I know that I'm being treated differently based on whether they perceive I come from a low-income community, or I am not a white American, or I'm an immigrant. …[T]hey treat you like they would treat an immigrant - which is not very nicely. (Mira, Graduate Student)*

James, an undergraduate, described his ongoing efforts to manage the stigma around his identity as a black man with a mental health condition. While his substance use disorder largely remains private unless he chooses to disclose, his race does not. James shared that he often attempts to “mask” his race to minimize the prejudice he encounters. He stated:

*I guess it, like, if I can hide being black… It's kind of weird because, like I'll try to hide my blackness if I can, you know? I try not to sound so ghetto ethnic when I'm speaking and different things of that nature. (James, Undergraduate Student)*

James noted that taking classes online provides him with one more tool that allows him to downplay stigmatized identities, including both his race and his mental health condition.

Michelle, a faculty person living with chronic illness, eloquently described the intersection of fatphobia with ableism, particularly in interactions with health care providers, stating:

…*[T]he weight gain was a symptom. [The doctor] saw the weight as the cause and couldn't get past that. And how weird that is to navigate, as someone who's chronically ill or disabled. When I'm sickest is when I'm thinnest and that's when I get treated the best. This is nonsense. And that obviously very much intersects with gender but it really intersects with race. BMI is a racist construct and a useless construct … I'm like, ‘Well, my weight's low because I haven't been able to eat in three months so maybe they'll take me seriously'. (Michelle, Faculty)*

This participant described the ways that her gender, race, and disability are all read through the lens of her body size, shifting the way healthcare providers respond to her other identities depending on her weight at the time.

As participants with multiple marginalized identities shared their experiences with ableism while receiving, providing, or training in healthcare, many of them commented on the inadequacy of institutional level efforts to improve diversity, equity, and inclusion. In particular, disability is often not included or considered an important part of diversity discussions. Several participants observed that diversity discussions at the university generally do not address intersectionality of different identities. Many highlighted that addressing diversity from only one perspective (such as race or non-conforming gender identities) is inefficient and leaves disabled students feeling left out. Several students argued that having only one identity in isolation be acknowledged and honored made diversity discussions feel superficial, if not hypocritical. A medical trainee stated:

*It would be great if the university really [took]a holistic approach, rather than putting us into buckets and saying: “Can the BIPOC part of you attend this focused activity, and then can the disabled part of you attend this other thing, and can the female gender identity attend this?” “Can you share your pronouns?” How much of this is just for show? We feel that way a lot. A lot of students like me with minoritized identities feel like a lot of this is for show rather than [establishing] genuinely safe places and true allyship. (Mira, Graduate Student)*

### COVID, surfacing ableism and expanding access: “The silver lining”

Study participants observed that the COVID-19 pandemic prompted employers, schools, and healthcare providers to more thoroughly and intentionally invest in infrastructure that generally increased access for all people, including those with disabilities. Faculty, staff and students living with disabilities reported that pandemic-inspired changes in policy and practices provided more flexible work schedules, created more options for professional engagement, and generated alternative modes of teaching and learning.

Students, in particular, described benefits generated from enhanced access to virtual or online engagement. One undergraduate explained that “*Taking classes remotely makes it actually easier for me to get captions from anywhere—and for a captioner to actually hear the professor.”* Another undergraduate noted that student-focused activities provided by places like the University's “D Center” (Deaf and Disability Cultural Center) was more easily able to serve a larger number of students across the tri-campus school: “*The D Center is only at the [main] campus, which is unfortunate, but luckily with COVID and stuff, we are able to do virtual events!”* Participants noted that remote learning did not, however, ameliorate notoriously long wait times for support services such as converting course materials to screen-reader accessible texts.

Participants also highlighted the ways in which the pandemic exacerbated challenges faced by disabled individuals living with multiply marginalized identities. Immigrant faculty and students were especially impacted by constantly changing policies as universities struggled to adapt to uncertain conditions. For students with disabilities living abroad, many felt like their circumstances were not being taken into consideration. One student stated:

*The latest exemption - in the spring, we were told that you need to commit to a date that you can reenter the US or resign, regardless of travel restrictions, regardless of embassy closures, regardless of global vaccine inequity, and this when low- and middle-income countries were not even giving vaccines at all. The university tries to be a global leader in public health, it's a domestic leader in the coronavirus response, and yet it talks from both sides of its mouth when it also tries to force its students to get back into the United States with no centering of the student experience. (Mira, Graduate Student)*

The pandemic also prompted public conversations about disability that brought ableism to the surface, providing critics and proponents alike opportunities to lay bare their arguments for pursuing disability justice or for defending the claim that some types of lives are more valuable than others. When asked for ideas on how to begin dismantling ableism in medical care, one faculty member stated:

*Yeah. I think one thing is tackling that part of ableism that says a disabled life is not worth living, which is super popular in movies and media, still, and is coming up. If we have to ration care for COVID, do we not give care to disabled people because their life isn't worth living? … No, life still has value and should be saved, right. So, challenging that giant part of eugenics and ableism… (Michelle, Faculty)*

Participants steeped in disability history highlighted direct connections between emerging discourses around rationing care during the pandemic and the U.S. Eugenics Movement that shaped the education and perspectives of our parents' and grandparents' generations.

Participants also reported that, in some cases, the shared experience of heightened illness awareness nourished newfound disability allyship, drawing the ideological underpinnings of ableist rhetoric to the surface of everyday discourses about disease, public health, and functional impairment.

*I think it's great that there's so much attention to post-COVID long haulers like me because it's getting people to understand, like you can't… you can't just will yourself better, you know? … I think there's also going to be a big sea change by the fact that there's going to be so many more disabled people… I feel like that's an extra burden that we bear, most of us, or at least the ones like me that are totally anxious and worried all the time. Like being judged differently or feeling like I have to work harder to make sure that nobody's feeling like I'm deficient, you know what I mean? Like I'm not pulling my weight or letting it affect my job performance and so it's been a huge relief to me to just know that like there are nice people in HR pulling for me and just in general, whether it's people you know, offering to give shared leave and…I would just like to think that we could reach that point eventually where it's - with everything related to disability or chronic illness - where it's kind of on [an even] plane, where we can talk about it comfortably, and it's not weird, and people can still offer support without crossing the line, you know... Again, I think* ***this might*** ***be...one of the rarer upsides to COVID, the silver lining****, I guess. (Jeanette, Staff)*

### Disability allyship and healthcare partnership building: “Work with me, not on me”

Despite ongoing ableist experiences in the healthcare system, participants highlighted the positive encounters they experienced during interactions with medical professionals. Collectively, participants felt more empowered in healthcare experiences involving active listening, practitioners taking time to understand and address individuals' priorities, and actions taken to ensure environments are accessible and inclusive. These experiences ranged from meeting individual access needs to creating universally designed spaces and interactions that benefit a wide variety of people. For example, one participant described the nuances of care in a provider-patient relationship and its impact on personal conceptualizations of health, noting,

*The biggest factor in a positive healthcare encounter that went well, was that I'm not only being cared for but that I'm cared about. Just on an almost personal level with the doctor. Like for example, I really like it when doctors are okay to just chat with me for a minute about anything or make jokes. …And, I think especially me personally, if I feel like I'm cared about, I feel better and I'm as healthy as I feel, right. And, I think it actually has positive impacts on whatever health issue that I'm dealing with at the time. (Eli, Undergraduate Student)*

Another participant described how important it was for her when providers communicate directly with her as an expert, especially having a childhood onset disability,

*I always feel it's positive when I feel like I'm really being listened to and my concerns are being taken into consideration…if the language is really accessible, especially because I was diagnosed when I was younger. So, the best experiences were when doctors would address me directly and would treat me as the patient and would listen to me about what I had to say and put me at the center of it so that they could understand from my position what's going on. Not just asking my parents questions about my health, because I was the expert on it, since it is my health. And so, like I've had doctors that have been amazing about it and have always looked me in the eye and addressed me and talk to me. And that was always the best because it made me feel like I was important and that I was actually the patient they were taking care of and that what I had to say mattered. (Jada, Undergraduate Student)*

Many other participants noted the importance of active listening, direct communication, and acknowledgment of expertise. For example,

*For me, a positive interaction with a healthcare provider is just the communication side of it. Are they listening to you? Are they hearing what you're asking or what you're telling them? My experience is that I know more about my disability than my healthcare provider because I live with it every day. That's true for all disabilities, but it's hard I think for healthcare providers to understand and always guide you in the right way. Healthcare providers that are really good, they listen because they know their limitations, and they know that they need to hear your side of it. (Justin, Faculty)*

Many participants also gave specific examples related to their disabilities, including being able to access free parking, accessible scheduling services, and quiet examination and treatment spaces. Others noted unconditional positive regard and support to ensure appropriate accommodations could be honored when they decided to disclose an invisible disability to their provider, as this participant described,

*The moment that I said anything about having a mental disability, which I don't think they had on file, she was like, ‘Oh okay. Do you need accommodations?' She was very responsive and understood, ‘Okay, that's important to you. You're telling me that. I need to understand and figure out what I can do for you.' (Amanda, Graduate Student)*

Participants also recalled moments when they felt their providers were partners in advocating for their needs, both in provider-patient communication as well as in communicating with other providers on their behalf,

*I destroyed my ankle and no one was taking me seriously about how bad the ankle injury was, and then I had a routine appointment with another provider who knew me, who knew I had had all these other ankle sprains that I have just ignored over the years…to see how bad it was [and advocate for me] like, “I'm calling the hospital and getting you into orthopedics tomorrow. This is not right.” And having someone who believed me, knew me, and knew that my tolerance for pain but it was like “this is unbelievable. What have they done?” And was realsly good with communicating with me and talking, “Well this is what the studies show,” and letting me have conversations at that science kind of level about decisions and care and making those choices. It felt like it was patient centered and I had a say and the power dynamics weren't as intense. (Michelle, Faculty)*

Ultimately, many participants described how positive interactions can still happen in healthcare while simultaneously acknowledging that the US healthcare system is “broken,” particularly for disabled people. One participant summarized,

*I had positive experiences when the specialists understood and listened to me even though the system was still broken. I loved when they used empowerment models that focuses on support and accommodations. I think that it was helpful when they have asked me what I would have liked to work on instead of just telling me what to do*. ***Work with me, not on me****. (Sung-Ho, Graduate Student)*

## Discussion

This qualitative, phenomenological study examined experiences of ableism and allyship of students, faculty, and staff on the University of Washington campus who identify as disabled/with a disability, d/Deaf, or as having a chronic health condition. The data and analysis presented here focused on participants' experiences in healthcare settings and generated four dominant themes: (1) Ever-present Ableism in Healthcare; (2) Ableism at the Intersections; (3) COVID: Surfacing Ableism and Expanding Access; and (4) Disability Allyship & Partnership Building. Despite increasing attention to DEI and emerging experiences of allyship and healthcare partnership, participants experienced frequent, shared experiences of ableism at individual, group/unit, and institutional levels, including healthcare encounters.

Sharing their experiences as patients, providers, trainees, or educators, participants described ableism as being ubiquitous in healthcare. Poorly planned attempts to build empathy backfired. Simulation activities, still present in many medical training contexts, frequently provide healthcare providers with overconfidence in their insight into a particular condition which can lead to minimizing or dismissing the actual lived experiences of patients (50–52). Particularly those who have lived many years with complex physical and psycho-social differences reported having their own expertise and knowledge frequently ignored (53). Having their lived experience minimized or dismissed by healthcare providers stymied timely access to medical options and led many patients to cycle through providers in search of adequate care. Though participants in this study did not describe healthcare avoidance *per se*, instead self-advocating by changing providers after ableist experiences, becoming educated in possible treatment options, or selectively seeking providers of a certain gender, for example, this did surface as a potential outcome for their peers during discussion. Current research supports the recognition of this potential outcome, describing how ableism and perceived discrimination among disabled people can lead to a reduction in healthcare-seeking behaviors or acceptance of limited treatment options presented by providers due to bias, thus contributing to greater health disparities and poorer health outcomes in this population (54–58). Additionally, this study's participants amplify previous studies' findings that subtle or overtly dehumanizing messages from healthcare professionals can contribute to internalized ableism (i.e., the shame, self-blame, and self-questioning) among people with disabilities (24, 59).

Participants who identify as both disabled and professionally embedded in the medical field expressed the general sentiment of being, as one staff member put it, “*shocked by how intolerant of chronic illness and disabilities most physicians really are.”* A medical school faculty member described health promotion for disabled people as infused with “*shaming and blaming”* rhetoric that “*is completely discriminating”* against those with disabilities. While these participants also described moments of allyship, medical providers themselves asserted that when one simply shows up in a healthcare context as a disabled person, many doctors automatically “*think of you as a train wreck.”* These findings mirror existing literature that highlights the ubiquitous presence of ableism in institutions of higher education, healthcare, as well as more broadly in society. For example, many studies have considered ableism in higher education, ranging from an examination of the tensions surrounding disclosure, to issues of physical or sensory inaccessibility, failure to provide rightful accommodations, and lack of resources for disability-related supports for students and faculty alike (34, 53, 60–62). Whether recipients of regular microaggressions or overt discrimination, participants described such experiences as the rule rather than the exception when recalling their campus experiences.

Interwoven into the stories of these participants were reflections on the ways in which discrimination based on gender, race, class, body size, and citizenship status intersect with ableism in medical contexts. Recognizing the ways that multiple axes of oppression work to complicate, exacerbate, or underpin each other is a critical first step toward untangling effective standards of medical provision and training from entrenched norms in healthcare settings that are in fact ineffective, discriminatory, and harmful (63–65). The data presented here demonstrate that rooting out biases based on, for example, race or gender, will require simultaneous attention to eradicating bias based on disability status. Current efforts toward creating more equitable environments in medical education that do not explicitly acknowledge and address intersectional identities are largely seen as being “*for show rather than…true allyship.”*

True allyship was described by the participants to a lesser extent. Nevertheless, when participants reflected on moments of disability allyship in healthcare contexts, the theme of partnership repeatedly emerged, or as one participant succinctly stated: “*work with me, not on me*.” Although comparatively rare, these experiences served as meaningful and important instances of solidarity and access that, taken together, can serve as a roadmap in effectively implementing inclusive practices within healthcare culture, whether formal or informal. This paucity of allyship experience is also reflected by a general lack of discussion of disability allyship in both scholarly literature and mainstream culture, though it is encouraging that this focus is growing more recently, especially as a result of the COVID-19 pandemic and concurrent social justice protests. Disabled scholars have addressed how being an ally, accomplice, or co-conspirator might be enacted, within film studies, disability studies, and more recently, in rehabilitation, through actions such as ensuring physically and sensorily accessible experiences and built environments, recruiting disabled students and hiring disabled faculty or clinical staff, and ensuring representation of actual disabled people in media while simultaneously avoiding pity or inspiration narratives (26, 27, 29, 31, 33, 66).

So, how do we use existing resources and new information from this study to further concretize allyship in rehabilitation, and move from theoretical examinations to translational impact? Forber-Pratt and colleagues lay out a valuable guideline for “showing up” as an ally, and offer strategies for both disabled and non-disabled identifying rehabilitation professionals (29). For non-disabled professionals, these include (1) understanding intersectionality; (2) asking and respecting choice of language; (3) embracing principles of universal design; (4) acting as an ally; (5) recognizing inspiration and overcoming narratives of disabled people; and (6) educating oneself on current disability rights issues faced by disability communities. For disabled professionals, this also includes checking internal disability-related biases and embracing cross-cultural disability solidarity (29).

**Figure 3:**
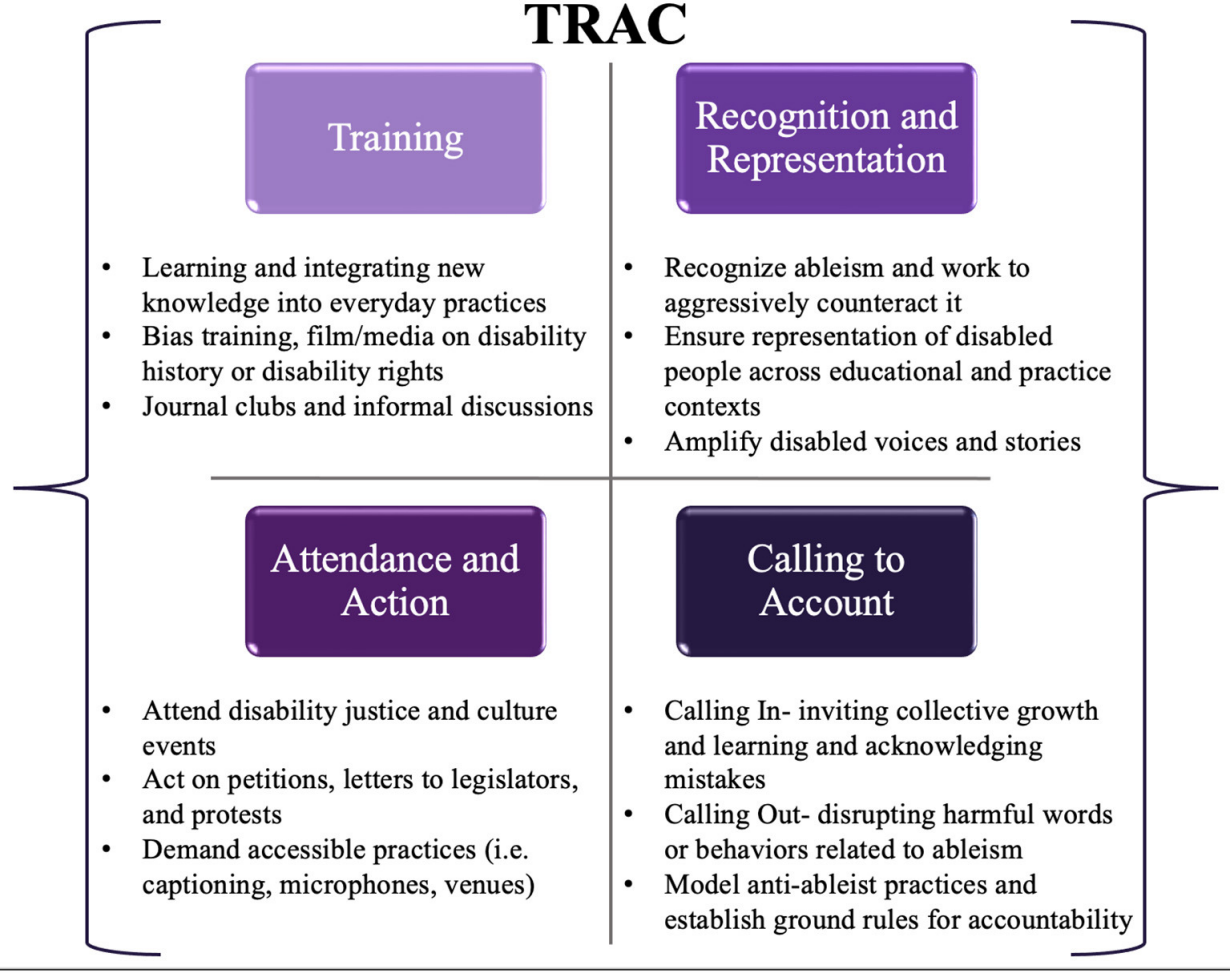
TRAC mnemonic for engaging in disability allyship: Training, Recognition and Representation, Attendance and Action, and Calling to account.

Using results from this study to amplify disabled voices in our campus and healthcare communities, we formulated additional considerations, in partnership with the UW disability community, to specifically address concept (4) above: acting as an ally. To operationalize these ideas, the research team devised the mnemonic TRAC, which includes Training, Recognition and Representation, Attendance and Action, and Calling to account (see Figure 3).

“Training” within the TRAC mnemonic involves a dedicated effort toward not only learning but integrating education into everyday actions. This includes educating oneself on the history of disability in the United States, the disability rights movement and subsequent legislation on disability, disability identity and diversity, intersectionality, accessibility tools and practices that are freely available, and learning about disability culture and pride. This could proceed formally through coursework or seminar series, participating in employer-offered bias training (which is increasingly mandated as a part of employer hiring processes), or informally through podcasts, TED talks, or documentaries such as the 2019 feature film, *Crip Camp: A Disability Revolution*, which are freely available on streaming services. The action component of training could be envisioned as a shift in everyday language used, creating a journal, book, or media club discussion, or simply having a conversation about issues raised by these training materials in professional, educational, or personal circles.

The R in TRAC consists of “Recognition and Representation.” First, rehabilitation trainees and professionals must recognize what ableism is, understand its widespread presence and influence, and actively work to counteract it. This could take the form of participating in an implicit bias association test, or reflecting on one's own personal experiences, including how positionality and bias may impact research production (43). It is important to recognize that whether through the lens of lived disability experience or through the lens of allyship, these experiences are biased by individual privilege and identity as well as experiences in society *because of* that privilege and identity, that both limit and shape our understanding of disability as a construct. Second, it is critical to acknowledge and actively work to increase disability representation in all aspects of society, including matriculated students in rehabilitation, their faculty, and within rehabilitation professions. Perhaps this might take place through review of admissions processes, active recruitment of disabled faculty and staff, or ensuring disabled community members are advising or participating in course construction, curriculum development, or as paid guest lecturers. Finally, ensuring representation is meaningful and avoiding tokenism is critical—rather than simply “checking a box,” recognizing that representation matters and is a key component of improving diverse, equitable, and inclusive educational and professional cultures for everyone.

The A in TRAC represents “Attendance and Action.” Attendance may be demonstrated by being present, whether virtually or in person, at advocacy events, or through other action such as petitions and letters or calls to legislators in support of policy and practice that is inclusive of disabled people. Action might manifest as holding space for a colleague who takes a bit longer to gather their thoughts and express their opinion in the middle of a meeting with a packed agenda. It might mean acknowledging contributions or ideas of a colleague or student who may be talked over or have their ideas co-opted by someone else in a more powerful social position. Or, this might mean attending disability focused gatherings, lectures, or events in the region, including municipal Americans with Disabilities Act (ADA) planning meetings, arts and culture events, public lectures, or rallies. Individual and collective action represent another important avenue of allyship. For example, automatically using accessible documents, microphones, requiring captions on videos in class, using image descriptions and alt text during professional presentations, and providing course content and assessments using multiple formats- all principles of universal design for learning that promote improved access for disabled people, non-native language speakers, and different types of learners alike. On campuses or in community settings, this could also be as simple as parking bikes or scooters clear of blocking ramps or sidewalk pathways, or moving these hazards to the side if you witness this during your own commute. Finally, this could consist of routinely performing accessibility audits or asking group members about access needs (which is distinct from requesting disability disclosure, an action to universally avoid in solidarity with privacy and confidentiality boundaries of disabled people).

Finally, the C in TRAC represents “Calling to account” – both through calling in (i.e., acknowledging a desire for collective growth and providing supportive space to make mistakes and learn), and, in some situations, calling out (i.e., directly challenging words or behaviors that are actively harmful to others). Calling to account is closely tied with action, but extends beyond action itself in that it holds our communities of education and practice accountable for those actions, and continually promotes a growth mindset that embraces tensions, successes, and “failing forward” to improve capacity for equity and inclusivity. Perhaps as a leader of a group or organization, this might look like inviting group members to engage in a discussion about current accessibility practices and future commitments (with deadlines) to improve access. As a course coordinator or instructor, this might be inviting students to come to class scent-free, or creating a collaborative list of alternative terms that could replace everyday ableist language that might come up in a classroom. As a professional, this could be modeling and consistently using accessible introductions that include image descriptions, pronouns, positionality, or announcing yourself as the speaker in a group setting for individuals who may have visual impairments, or speaking at a more comfortable pace for ASL interpreters, as well as consistently engaging in access check-ins and explaining why it's important. This could be a joint crafting of a lab/course/program diversity or accessibility statement where everyone is involved in creating the culture and expectations for practicing inclusivity. Finally, while calling in is preferred, there are instances where it may be appropriate to call people out. These situations are ones in which the power differentials are so great, and in which there may not be an opportunity for an interactive dialogue, that calling someone out for ableist practices may be appropriate. However, in the majority of situations, there is an opportunity for dialogue, thus a process of calling in may be far more effective in fostering disability allyship.

## Strengths, limitations, and future directions

This study is one of the first of its kind in the US to use a CDS framework to understand lived experiences of ableism and allyship across faculty, staff, and students in a university setting, and to subsequently apply these experiences to inform the development of more inclusive practices in rehabilitation education in a post-COVID era. It builds on the foundational work of leaders in the field who have pioneered the application of a CDS approach in rehabilitation education and practice (16, 37–39). This perspective offers a more nuanced understanding of disability as a complex, relational identity that can broaden perspectives of trainees and professionals in the field. An additional strength is the diversity of the study participants as well as the research team. Disabled individuals were included in all aspects of the development and execution of the study procedures, and over one third of our participants also identified as disabled individuals of color. Further, our participant cohort included faculty, staff, and students; the shared experiences of ableism and allyship in healthcare across these groups despite unique roles and circumstances adds to the generalizability of our theoretical framework used to interpret the results. Another strength of this study was the sustained opportunity for engagement by participants, many of whom expressed their eagerness to participate further in the project after the initial phases. In conjunction with several participants of our focus groups and interviews, the research team is engaged in ongoing curriculum development focused on disability allyship for rehabilitation students and professionals that is directly informed by our results. Not only are these participants engaged in active solution building, their impact will continue to be evident for disabled students, faculty, and staff that will come after them. Finally, the co-constructed research process has led to a concrete end product, TRAC, that provides a roadmap for action for students, faculty, and professionals in clinical settings who are committed to improving inclusivity, access, and celebration of disability as diversity.

There are several limitations to this study as well. First and foremost, given the roles of the research team as disability studies and rehabilitation scholars, there was the potential for acquiescence bias among our participants. This was mitigated by exhibiting unconditional positive regard, frequently reminding participants that unique and contrasting perspectives were valued. Further, by carefully curating and sharing working definitions of disability, ableism, and allyship as well as interview questions with our participants prior to the discussion, the research team may have unduly influenced participants' thinking about these concepts that may have been expressed differently if these materials were not shared. However, the research team felt strongly that this was not only a procedure that would maximize accessibility and reduce potential response anxiety, but also allow participants to think deeply about these complex topics and come prepared to the discussions. An additional limitation is that though the responses have theoretical generalizability, the experiences represented in this study only represent a small sample at a single institution, and cannot be more broadly generalized to other disability communities. However, the saturation of responses across a diverse group indicates that this data is important for informing future work. A final limitation of this study includes the potential for selection bias. Highly educated, well-resourced disabled students, faculty, and staff self-selected to participate, and those interested in sharing their experiences of ableism and allyship may have stronger self-advocacy backgrounds and lower instances of internalized ableism than other disabled individuals who may have chosen not to participate.

Results from this study are currently being incorporated in the crafting of a disability allyship training curriculum based on the lived experiences of disabled students, staff, and faculty on UW campus. Future work will include delivery of this bespoke training curriculum to groups/units within rehabilitation and other medical programs, as well as in non-healthcare related programs across campus. Curriculum delivery will be evaluated using pre-post analysis of attitudes toward disability, inclusive practice implementation, and formal training feedback opportunities. In the future, it will also be critical to extend this line of inquiry to rehabilitation clinicians who are already embedded in the field, and who may have longstanding views of disability, ableism, and allyship that may differ from students or faculty in university settings. Finally, further research is warranted to understand and expand educational access methods and tools (such as online learning) that can positively impact individuals with stigmatized identities within rehabilitation and beyond.

## Conclusion

Rehabilitation students, educators, and practitioners may not be aware of how ableism affects their peers with disabilities or underpins professional education and clinical practice. A CDS framework provides a valuable lens through which to examine meanings of disability and allyship and how these concepts are operationalized in the context of rehabilitation education and practice. Foregrounding the lived experiences of disabled individuals within campus and healthcare culture must be central to efforts to identify, eliminate, and prevent systemic ableism and, in turn, advance inclusivity. Specifically infusing disability allyship into DEI initiatives within rehabilitation education, practice, and beyond will prepare students and existing professionals to more proactively address disability-related disparities in the post-pandemic era.

## Data availability statement

The datasets presented in this article are not readily available because of confidentiality and privacy considerations for our participants. Requests to access the datasets should be directed to hfeldner@uw.edu.

## Ethics statement

The studies involving human participants were reviewed and approved by University of Washington Human Subjects Division. Written informed consent for participation was not required for this study in accordance with the national legislation and the institutional requirements.

## Author contributions

HF, HE, MH, and DL led the data coding, thematic discussion of data analysis, and participated in draft editing and review. HF and HE led the manuscript preparation and writing and were responsible for creating the TRAC approach to disability allyship. All authors participated in securing funding, as well as the planning and execution of the study, including study coordination, data collection and preparation, and data analysis.

## Funding

This work was supported by funding from the University of Washington Center for Leadership and Innovation in Medical Education (CLIME) Competitive Small Grants Program, and the UW Center for Research and Education on Accessible Technology and Experiences (CREATE).

## Conflict of interest

The authors declare that the research was conducted in the absence of any commercial or financial relationships that could be construed as a potential conflict of interest.

## Publisher's note

All claims expressed in this article are solely those of the authors and do not necessarily represent those of their affiliated organizations, or those of the publisher, the editors and the reviewers. Any product that may be evaluated in this article, or claim that may be made by its manufacturer, is not guaranteed or endorsed by the publisher.
