# Supplementary material for: Infusing disability equity within rehabilitation education and practice: A qualitative study of lived experiences of ableism, allyship, and healthcare partnership
Source: Front Rehabil Sci. 2022 Aug 2;3:947592. doi: 10.3389/fresc.2022.947592 (PMC9397845; doi:10.3389/fresc.2022.947592)
Supplement: Supplementary file 1 [file Data_Sheet_1.PDF]

COREQ (COnsolidated criteria for REporting Qualitative research) Checklist

A checklist of items that should be included in reports of qualitative research. You must report the page number in your manuscript where you consider each of the items listed in this checklist. If you have not included this information, either revise your manuscript accordingly before submitting or note N/A.

Topic	Item No.	Guide Questions/Description	Reported on Page No.
**Domain 1: Research team and reflexivity**			
Personal characteristics			
Interviewer/facilitator	1	Which author/s conducted the interview or focus group?	P5L192
Credentials	2	What were the researcher's credentials? E.g. PhD, MD	P1L4-6
Occupation	3	What was their occupation at the time of the study?	P1L4-11
Gender	4	Was the researcher male or female?	P4L151
Experience and training	5	What experience or training did the researcher have?	P4L150-155; P5L211-213
Relationship with participants			
Relationship established	6	Was a relationship established prior to study commencement?	P5L193-195
Participant knowledge of the interviewer	7	What did the participants know about the researcher? e.g. personal goals, reasons for doing the research	P5L202-208
Interviewer characteristics	8	What characteristics were reported about the inter viewer/facilitator? e.g. Bias, assumptions, reasons and interests in the research topic	P5L207-208
**Domain 2: Study design**			
Theoretical framework			
Methodological orientation and Theory	9	What methodological orientation was stated to underpin the study? e.g. grounded theory, discourse analysis, ethnography, phenomenology, content analysis	P4L139-145
Participant selection			
Sampling	10	How were participants selected? e.g. purposive, convenience, consecutive, snowball	P4L165-168
Method of approach	11	How were participants approached? e.g. face-to-face, telephone, mail, email	P4L164-165
Sample size	12	How many participants were in the study?	P7L262
Non-participation	13	How many people refused to participate or dropped out? Reasons?	P7L266
Setting			
Setting of data collection	14	Where was the data collected? e.g. home, clinic, workplace	P5L183
Presence of non-participants	15	Was anyone else present besides the participants and researchers?	P5L192-193
Description of sample	16	What are the important characteristics of the sample? e.g. demographic data, date	P5L262-265
Data collection			
Interview guide	17	Were questions, prompts, guides provided by the authors? Was it pilot tested?	P5L202-208
Repeat interviews	18	Were repeat inter views carried out? If yes, how many?	P5L183-184
Audio/visual recording	19	Did the research use audio or visual recording to collect the data?	P5L198-199
Field notes	20	Were field notes made during and/or after the inter view or focus group?	P5L196-197
Duration	21	What was the duration of the inter views or focus group?	P5L184
Data saturation	22	Was data saturation discussed?	P6L249
Transcripts returned	23	Were transcripts returned to participants for comment and/or	P6L256-259

Topic	Item No.	Guide Questions/Description	Reported on Page No.
		correction?	
**Domain 3: analysis and findings**			
Data analysis			
Number of data coders	24	How many data coders coded the data?	P6L250-251
Description of the coding tree	25	Did authors provide a description of the coding tree?	P6L256
Derivation of themes	26	Were themes identified in advance or derived from the data?	P6L251-252
Software	27	What software, if applicable, was used to manage the data?	P6L248-249
Participant checking	28	Did participants provide feedback on the findings?	P6L256257
Reporting			
Quotations presented	29	Were participant quotations presented to illustrate the themes/findings? Was each quotation identified? e.g. participant number	P7-14, L288-556
Data and findings consistent	30	Was there consistency between the data presented and the findings?	Throughout
Clarity of major themes	31	Were major themes clearly presented in the findings?	P6L266-268; P14 L560-563
Clarity of minor themes	32	Is there a description of diverse cases or discussion of minor themes?	N/A

Developed from: Tong A, Sainsbury P, Craig J. Consolidated criteria for reporting qualitative research (COREQ): a 32-item checklist for interviews and focus groups. *International Journal for Quality in Health Care*. 2007. Volume 19, Number 6: pp. 349 – 357

Once you have completed this checklist, please save a copy and upload it as part of your submission. DO NOT include this checklist as part of the main manuscript document. It must be uploaded as a separate file.
