# Supplementary material for: Infusing disability equity within rehabilitation education and practice: A qualitative study of lived experiences of ableism, allyship, and healthcare partnership
Source: Front Rehabil Sci. 2022 Aug 2;3:947592. doi: 10.3389/fresc.2022.947592 (PMC9397845; doi:10.3389/fresc.2022.947592)
Supplement: Supplementary file 2 [file Data_Sheet_2.PDF]

Focus Group Questions

Welcome Participants

Introductions of Team: *Name, Pronouns, Role, Identity (how we interact with disability or act as an advocate), and fun fact*

Introduction of the participants: *(may remain anonymous, intro self how you feel comfortable- I.e. with name/pseudonym/screen name/role/fun fact)*

Purpose of the Study: *This study is being conducted by a team of researchers from Disability Studies, The D Center, and Rehabilitation Medicine. The purpose of this study is to understand experiences of both ableism or discrimination and allyship or inclusive community building across the UW campus and within healthcare experiences. Our aim is to develop a disability allyship training curriculum that is rooted in lived experiences and can be implemented in the education and training of healthcare professionals and others across the UW campus to improve our inclusive campus culture.*

Introduction of study and procedures: *We will be asking a series of questions to you today about these topics. There are no right or wrong answers, and we value everyone's diverse experiences. We will be audio recording our conversation, all names and places mentioned will be changed and the recordings will be destroyed following transcription. After turning the recording on, we will ask for verification that you consent to participate in these recorded focus groups/interviews. At the conclusion of the focus groups, we will send you an electronic gift card for $30 as compensation for your time and expertise. During the focus group, you may use the chat room, hand raise feature, audio, or video to participate. These zoom features will be monitored by one of the discussion leaders. You may choose to keep your camera on or off, and we ask that you please mute your microphone unless you are speaking. You are free to leave the interview at any point and are not obligated to answer any questions you are not comfortable with.*

Operational Definitions:

We will be using several key terms today, such as disability, ableism, and allyship. We want to acknowledge that these terms are complex, and their definitions are nuanced and often contested. We all come to this table with different understandings and lived experiences, so we felt that discussing these terms prior to our questions would assist having a fruitful shared discussion. We respect your individual conceptualizations and definitions of these terms and how they shape your experiences and identities. We value you joining us today and are eager to hear your perspectives during our time together.

Disability:

Disability is understood to arise from the interaction between a person's health condition or impairment and the multitude of influencing factors in their environment. Many people also refer to themselves as a person with a disability, being disabled, having impairments or health conditions, or by a specific medical diagnosis or cultural group such as autistic, blind, D/deaf, linguistic minority. It is important to recognize that many people believe that disability is NOT a trait or attribute of the individual; rather, disability is experienced as a result of levels of structural oppression based on ideologies that denigrate 'disability'. Many disabled people attribute external prejudice and denigration in social and cultural spheres as one of the reasons they experience 'disability'. They challenge the idea of 'typical functioning' of both the body and mind as a measure of ability or inability. The failure of society to recognize differences in functioning of the body/mind as both natural and neutral is the root cause of 'disability'. Disability intersects and is connected to all forms of structural oppression such as racism and sexism, especially those rooted in Western ideologies of white patriarchal supremacy and colonial histories of domination. Disabled people experience disability in different ways depending on other aspects of their identity and lived experience. It is acknowledged that the study of disability, and historically disability research, is also rooted in these structures of power.

Ableism:

Ableism is a set of assumptions and practices promoting the differential or unequal treatment of people because of actual or presumed non-typical functioning (i.e., disability). Ableism takes many forms, including non-disabled people controlling disabled people's narratives, judgements on the reality and quality of disabled people's lives, and assumptions that disability is static or unchanging. It is important to note that Ableism is NOT just directed at bodies/minds with physical impairments/and "apparent" differences. People with non-apparent impairments navigate ableism in both similar and different ways. Through this research project, we are hoping to explore the many ways ableism affects individuals with

chronic illness, psychiatric impairments (mental illness), learning impairments, physical impairments, and internalized ableism.

Allyship:

Allyship is a process rather than a singular concept; [allyship] consists of a series of actions, and approaches that attempt to recognize, mitigate and challenge structural forms of oppression towards specific communities within both interpersonal interactions and in systemic changes to existing power inequalities. Practicing allyship with and for disabled people/PWD is continually checking assumptions about what informs your views about disability and ability. Some of the ways allyship towards the disability community is practiced is by:

- Listening to disabled people's stories
- Educating yourself about disability and current issues that impact their communities
- Checking assumptions about who is non-disabled/disabled
- Challenging cultural narratives that reinforce ideals of normality
- Identifying and challenging ableist terminology

Disclosure

We want to be clear that we are not requesting disclosure of specific disability or impairment-related details. If you feel this is important to share when discussing your experiences, you may do so at your own comfort level, but we will not be categorizing any responses by disability or impairment details.

Section 1: Ableism/Discrimination (Social, Physical, Cultural)

1. Can you share with us some interactions/situations that have you encountered on the UW campus where you feel ableism was a part of the experience?
    a. How did you respond and/or what actions did you take?
    b. Can you share specific experiences that involved people in positions of power (I.e. administrators, faculty, supervisors)?

2. Can you share your insights into why you think you had these experiences of ableism?

3. In what ways do you think ableism can be challenged at the UW?
    a. On the macro level? (Systems, policies etc.)
    b. On the micro level? (Perspectives, beliefs, prejudice)

Section 2: Healthcare/Ableism- (general experiences)

We know that many people have faced or are facing discrimination and ableism within the US healthcare system. Because part of the purpose of this study is to train future healthcare providers to be better allies, this next set of questions focuses broadly on your healthcare experiences, both positive and negative. We will not ask you to share specific details of your health or healthcare encounters, but rather we are asking you to think about how those experiences can be used to create actionable change in healthcare education.

1. If you think about a healthcare encounter that went well in your mind, what features or factors were in place to make that experience a positive one? (I.e. environment, interpersonal interaction, communication, scheduling, transportation, access to needed equipment, etc.)

2. If you think about a healthcare encounter that went poorly in your mind, what features or factors were in place to make that experience a negative one?

3. What needs to change in healthcare and in medical education to better support people with disabilities and create more inclusive environments/interactions?

Section 3: Allyship/Community Support

1. What are the ways in which your personal experiences related to disability and identity are acknowledged and honored within the UW Campus culture?

2. From your perspective what are some ways students, staff and faculty can practice allyship towards the disability and/or D/deaf community at the UW?
   a. Can you discuss specific actions on the macro level? (Systems, policies, architecture, etc. at institutional/organizational level)
   b. Can you discuss specific actions on the micro level? (Perspectives, beliefs, prejudice in classrooms, campus spaces, events, etc.)
